# Stage-specific regulation of seed germination under saline-alkali stress and implications for sequential emergence capacity

**DOI:** 10.3389/fpls.2026.1943637

**Published:** 2026-09-04

**Authors:** Weiyu Xiong, Xinran Li, Yezi Zhang, Jingyu Wang, Siqi Ge, Dali Liu, Wang Xing

**Affiliations:** 1 National Beet Medium-Term Gene Bank, Heilongjiang University, Harbin, China; 2 Key Laboratory of Beet Genetics and Breeding/College of Modern Agriculture and Ecological Environment, Heilongjiang University, Harbin, China; 3 China National Seed Group Co., Ltd., Sanya, China

**Keywords:** saline-alkali stress, seed germination, stage specificity, sequential emergence capacity (SEC), regulatory mechanisms

## Abstract

Soil salinization and alkalization severely constrain crop emergence and stand establishment. The seed germination stage involves rapid transitions through imbibition, metabolic reactivation, radicle protrusion, and early seedling establishment. Its responses to saline-alkali stress are stage-specific rather than simply representing an early manifestation of seedling-stage saline-alkali tolerance. This review synthesizes research progress on seed germination under saline-alkali stress, with emphasis on distinguishing the differential constraints imposed by neutral salts versus alkaline salts on germination. It summarizes regulatory mechanisms including early perception, ion and redox homeostasis, autophagy and proteostasis, cell-wall biomechanical remodeling, and the ABA-GA-mediated balance between growth and defense. On this basis, we propose sequential emergence capacity (SEC) as a conceptual framework for process-oriented phenotyping and evaluation. It covers the sequence from early damage buffering to germination progression and early establishment, and includes cross-stage recovery or re-exposure responses when relevant. SEC comprises four dimensions: damage avoidance capacity (DAC), germination progression robustness (GPR), early establishment quality (EEQ), and cross-stage adaptive potential (CAP). DAC, GPR, and EEQ constitute the operational core (SEC_core); CAP is retained only as an exploratory or mechanistic validation dimension. In practice, SEC is meant to be used primarily as a multidimensional phenotypic profile. Composite scores are optional and, if used, must be validated against substrate- or field-level emergence data. Future efforts should develop stepwise, high-throughput phenotyping pipelines based on SEC_core. These could help uncover the genetic control of emergence-related traits, if validated under field conditions, and thereby bridge the gap between laboratory assays and field performance in saline-alkali environments.

## 1 Introduction

Soil salinization and alkalization are major constraints on agricultural production and ecological restoration. According to the FAO (2024), approximately 1.381 billion hectares of land worldwide are currently salt-affected. The mechanistic basis of plant tolerance to saline-alkali stress, including ion homeostasis, osmotic regulation, reactive oxygen species (ROS) scavenging, and related signaling networks (Cao et al., 2022), has been studied extensively, but mostly in the seedling stage and vegetative growth. In agronomic practice, however, the main challenge on saline-alkali soils is unstable emergence and poor stand establishment, which compromise all subsequent growth. Some germplasm that shows moderate tolerance under controlled conditions often fails to maintain a consistent emergence advantage in the field (Ganie and Azevedo, 2026), suggesting that the germination stage is subject to constraints distinct from those at later developmental phases.

This difference originates from the physiological characteristics of the germination stage. Dry seeds initiate germination from a highly dehydrated glassy state and must complete imbibition, membrane system repair, respiratory reactivation, storage reserve mobilization, and radicle protrusion within a narrow time window. At this point, photosynthesis and a functional root system are absent, so defense and repair processes rely entirely on limited internal reserves (El-Maarouf-Bouteau, 2022). More critically, selective membrane permeability and ion extrusion capacity are not yet fully restored during early imbibition (Hoai et al., 2023), while external salts have already entered the tissues along with water. As a result, ionic imbalance, osmotic stress, and secondary oxidative damage may occur at a very early stage (Hasanuzzaman, 2025). Within this vulnerable window, the effects of salt stress and alkali stress are not identical. Neutral salts (mainly NaCl and Na_2_SO_4_) act primarily through Na^+^ overaccumulation, K^+^ depletion, osmotic imbalance, and elevated ROS levels across multiple growth stages (Hualpa-Ramirez et al., 2024), whereas alkaline salts such as Na_2_CO_3_ and NaHCO_3_ introduce additional constraints including high pH, reduced nutrient availability, and deteriorated soil properties (Li and Yang, 2023). For germinating seeds, whose membrane potential and proton-driven transport have not yet been reestablished, these high-pH-related effects represent more than merely an intensification of salt injury.

**Figure 1:**
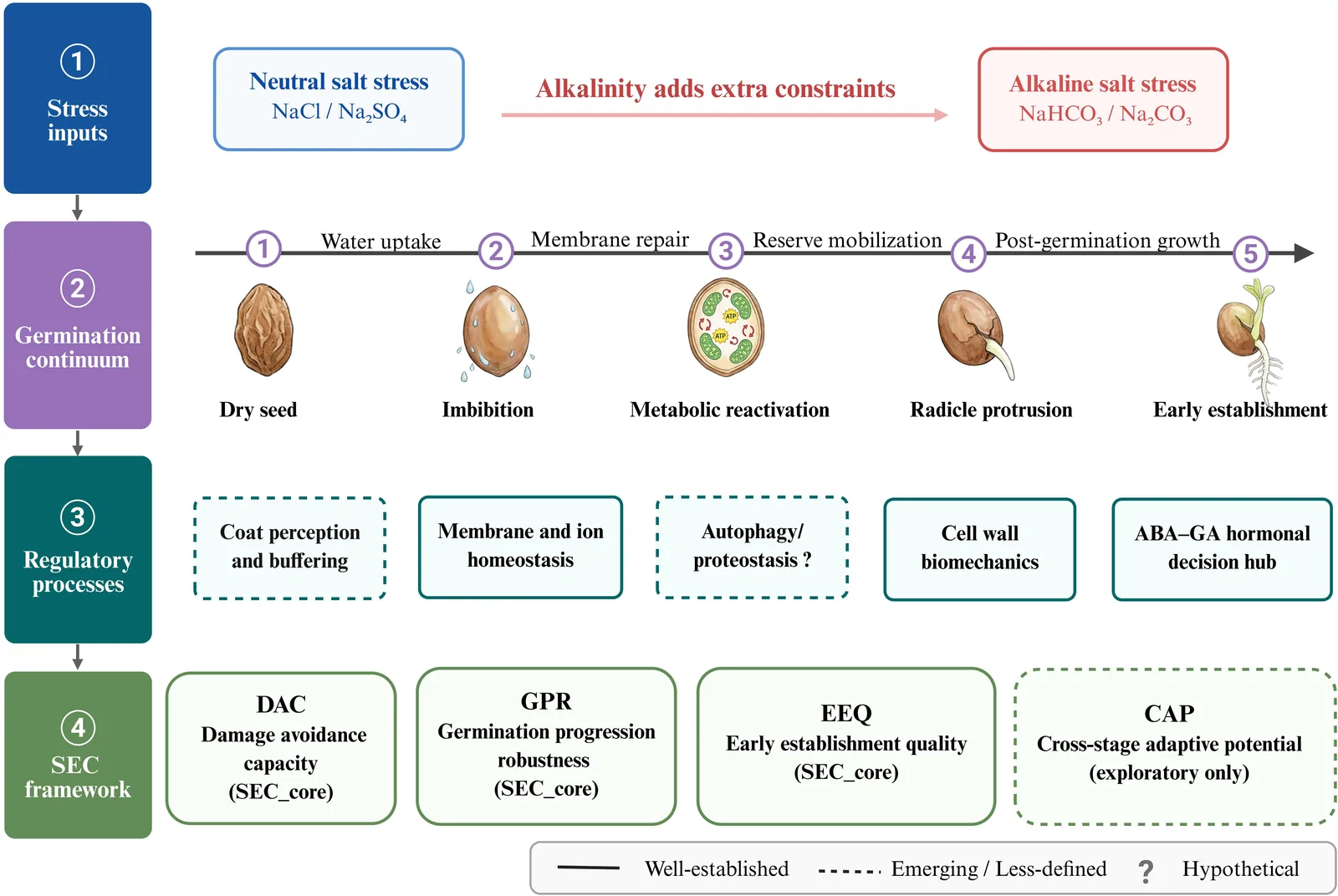
From saline-alkali stress inputs to the SEC framework during seed germination. Neutral salt stress and alkaline salt stress are depicted as external inputs; alkalinity imposes additional constraints beyond neutral salts. These inputs are mapped onto a simplified germination continuum (dry seed → imbibition → metabolic reactivation → radicle protrusion → early establishment). High-level regulatory processes include seed-coat-mediated perception and buffering, membrane and ion homeostasis, autophagy/proteostasis (emerging), cell-wall biomechanics, and the ABA-GA decision hub. The lower tier summarizes the proposed sequential emergence capacity (SEC) framework: SEC_core = {DAC, GPR, EEQ}; CAP is exploratory only. Solid outlines indicate better-supported elements; dashed outlines and question marks indicate emerging or less-defined components. The schematic is conceptual; timing and relative importance vary with species, stress regimes and experimental systems. SEC is presented as a process-oriented phenotyping framework complementary to final GP and vigor indices, not as a new molecular tolerance mechanism.

Against this background, this review treats saline-alkali performance at the germination stage as a stage-specific process with its own physiological logic, and organizes the discussion around phases: stress perception, signal transduction, homeostatic reestablishment, growth decision, and cross-stage integration. We further propose sequential emergence capacity (SEC) as a complementary, process-oriented framework for emergence under saline-alkali stress (**Figure 1**). SEC is not intended as a new biological concept, nor as a replacement for germination percentage (GP), germination energy (GE), or conventional vigor indices. Instead, SEC emphasizes that emergence is a time-ordered sequence: rather than relying on a single endpoint, it asks whether seeds can limit early injury, progress stably to radicle protrusion, and convert protrusion into viable early establishment.

## 2 Distinct constraints of neutral vs. alkaline salts

In this review, we classify stress into four categories: (i) Salinity refers to stress dominated by neutral salts (commonly NaCl and/or Na_2_SO_4_) with osmotic and ionic effects (Fang et al., 2021); (ii) Alkaline-salt stress is imposed in solution experiments by salts such as NaHCO_3_ and Na_2_CO_3_, which combine ionic and osmotic effects with bicarbonate or carbonate chemistry and elevated pH (Li and Yang, 2023). (iii) Alkalinity or high-pH stress applies when pH elevation is the factor of interest, and should be supported by appropriate controls (Raja et al., 2026). (iv) Soil sodicity involves excessive exchangeable sodium and the associated physical constraints, including clay dispersion, structural degradation, surface crusting, reduced infiltration, poor aeration, and increased mechanical resistance to emergence (Maurya et al., 2022). Unless otherwise specified, the experimental contrasts discussed here refer to conditions (i)–(iii) in germination media, not to the full edaphic syndrome of sodic field soils. Inferences about stand establishment on sodic soils therefore require additional caution beyond solution-based germination assays.

**Figure 2:**
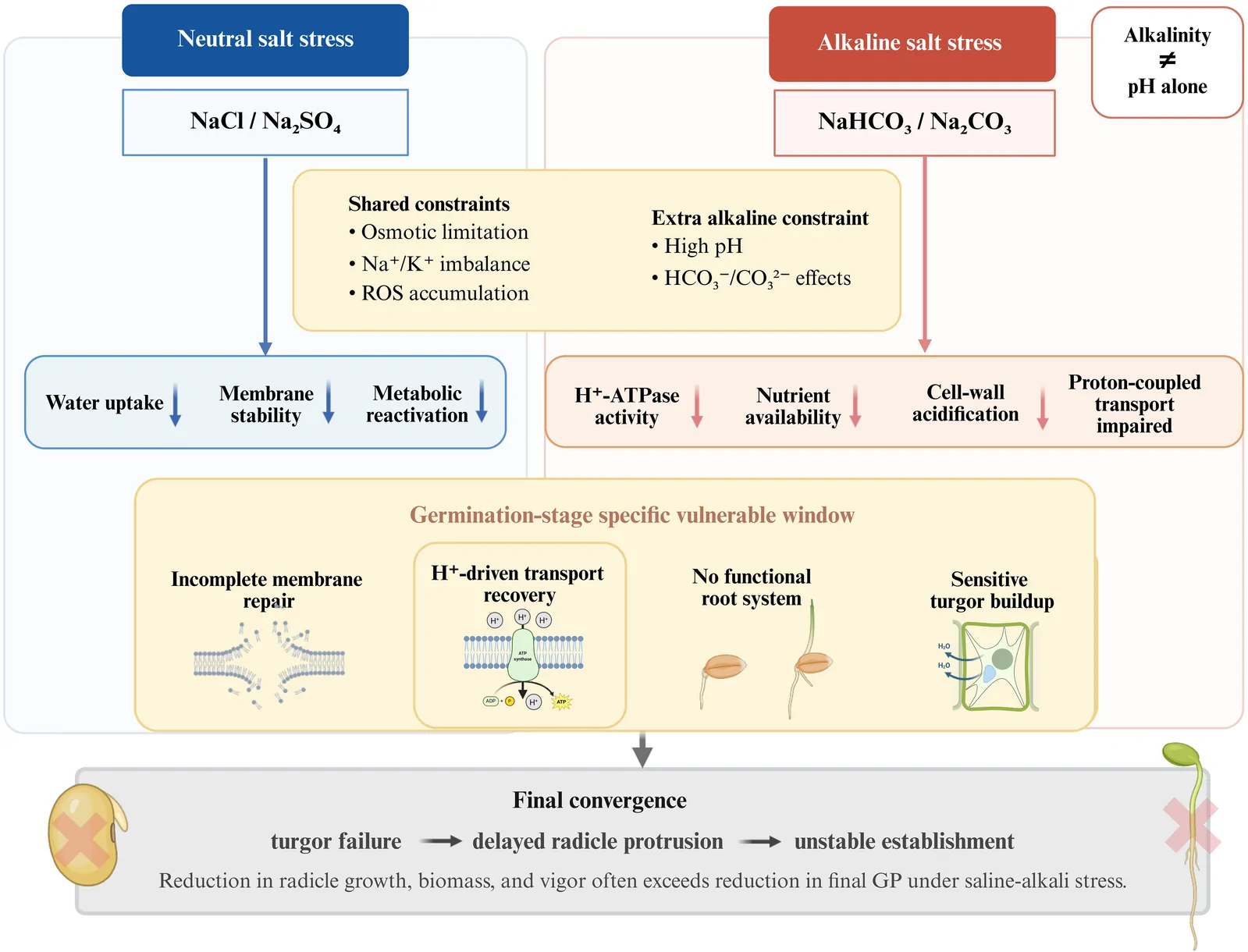
Differential constraints imposed by neutral and alkaline salts during seed germination. Neutral salts, represented here by NaCl and Na_2_SO_4_ primarily impose osmotic limitation, Na^+^/K^+^ imbalance, and ROS accumulation, leading to reduced water uptake, impaired membrane integrity, and delayed metabolic reactivation. Alkaline salts, represented by NaHCO_3_ and Na_2_CO_3_ share these constraints but additionally impose high-pH- and carbonate/bicarbonate-related effects, including disrupted H^+^ gradients, reduced nutrient availability, impaired cell-wall acidification, and altered proton-coupled transport. These inputs act upon a germination-stage vulnerable window (incomplete membrane repair, ongoing recovery of H^+^-driven transport, lack of a functional root system, and sensitive turgor buildup) and converge on turgor failure, delayed radicle protrusion, and unstable early establishment. Reductions in early growth and vigor often exceed those in final GP. The schematic is comparative and conceptual; relative weights vary with species, salt composition, concentration, pH, and system.

During germination, both neutral and alkaline salts can cause osmotic stress, ionic toxicity, and secondary oxidative damage and membrane injury, as manifested by reduced imbibition, delayed germination, and inhibited radicle elongation (Yang et al., 2019). Saline-alkali stress, however, is not a homogeneous treatment. Even among neutral salts, different salt types vary markedly in their inhibitory effects on germination (Berti et al., 2025). In actual saline-alkali fields, particularly in soda saline-alkali soils, seeds are more often exposed to high-pH systems containing NaHCO_3_ and Na_2_CO_3_ rather than to NaCl alone (Yang et al., 2019; **Figure 2**). Caution should therefore be exercised when extrapolating germination performance under NaCl treatments to saline-alkali field conditions.

The key difference between alkaline salts and neutral salts lies in the additional effects of high pH and HCO_3_^-^/CO_3_²^-^, beyond the salt and Na^+^ effects common to both types (Sharma et al., 2024). Studies in various species, including *Brassica oleracea* var. *capitata* (Wang et al., 2025b), *Physocarpus amurensis* (Liu et al., 2021), *Origanum vulgare* (Yu et al., 2024), and *Andrographis paniculata* (Zhang et al., 2026b), have consistently shown that NaHCO_3_ or Na_2_CO_3_ generally has a stronger inhibitory effect on germination than NaCl or Na_2_SO_4_ Moreover, *Brassica oleracea* var. *alboglabra* responded differently to salt versus saline-alkali treatments even under the same Na^+^ concentration (Li et al., 2026), indicating that the addition of alkaline components and the accompanying pH increase impose effects beyond a simple extension of NaCl toxicity. Component-resolved interpretation, however, requires transparent matching of Na^+^, osmotic potential and ionic strength, clear reporting of anion identity and single-salt versus mixture designs (Munns and Tester, 2008), and, where high-pH effects are claimed, pH controls that are not confounded by carbonate chemistry. Equimolar salt comparisons alone do not guarantee equivalent Na^+^ or osmotic load.

This difference is related to the physiological state of seeds during germination. Following imbibition, seeds must rapidly accomplish membrane repair, ion redistribution, and the establishment of turgor required for radicle protrusion. High pH may interfere with plasma membrane H^+^-ATPase-dependent processes, compromise the restoration of transmembrane electrochemical gradients, and affect nutrient availability as well as the conditions required for cell wall acidification and loosening (Cao et al., 2022; Li et al., 2022). These effects are likely more pronounced in seeds that have not yet developed a functional root system. In *Avena sativa*, GP, GE, and germination index (GI) were influenced not only by salt concentration but also by pH and the proportion of alkaline salts (Ahmed et al., 2023). In *Panicum miliaceum*, increasing salt concentration and pH jointly constrained germination and early establishment, with alkaline salts showing stronger inhibition than neutral salts (Xiao et al., 2026b). A cautious interpretation is that high pH contributes to and exacerbates alkaline salt injury, but the effect of alkaline salts cannot be reduced to a simple pH effect alone.

This notion is supported by studies conducted at post-germination stages. In *Arabidopsis*, neutral and alkaline salts at equal concentrations elicit overlapping responses, but alkaline salts exert more distinctive effects on cell wall remodeling, hormone networks, and iron utilization. The cell wall-associated β-galactosidase BGAL4 was induced under neutral salt but not under alkaline salt; alkaline salts also caused stronger disruption of auxin, ethylene, and jasmonate pathways, accompanied by reduced iron use efficiency (Almira-Casellas et al., 2024). In *Oryza sativa*, the cell wall protein OsGP1 participates in maintaining cell wall integrity and regulating ROS homeostasis under soda saline-alkali stress (Zhu et al., 2024). In blueberry, H^+^ influx and efflux were restricted at pH 8.0, with most plasma membrane H^+^-ATPase transcripts downregulated, whereas enhanced H^+^ pumping capacity improved alkali tolerance (Chen et al., 2022). Although these findings are derived mainly from post-germination stages, they indicate that the effects of high pH on the cell wall environment and proton gradients are independent and cannot be reduced to an enhancement of Na^+^ effects.

More direct evidence comes from water uptake dynamics and recovery studies. In *Medicago sativa*, NaCl primarily suppressed water uptake throughout the germination process, whereas NaHCO_3_ had less effect on early imbibition and mainly inhibited the growth-phase water uptake, as reflected by restricted radicle and plumule elongation. Simply raising the pH of NaCl solution did not fully replicate the damage caused by NaHCO_3_ and lowering the pH of NaHCO_3_ solution only partially alleviated the inhibition (Wang et al., 2025c). This indicates that alkaline salt injury is not equivalent to NaCl effects plus high pH. On the other hand, recovery of germination varied across species and treatments: *Oryza sativa* (Jin et al., 2020) and *Halogeton arachnoideus* (Zhao et al., 2021) showed some recovery after relatively strong saline-alkali stress, whereas *Triticum aestivum* (Li et al., 2024) exhibited markedly lower germination recovery under combined alkaline salt treatments than under neutral salt treatments. These observations suggest that the proportion of delayed germination versus impaired viability differs among experimental systems, and that salt tolerance mechanisms are not directly equivalent to saline-alkali tolerance mechanisms.

## 3 Sensing, signaling and homeostasis

### 3.1 Early perception at the seed coat and membrane

Early responses to salt stress during germination are not mediated by a single receptor, but involve the seed coat/mucilage interface, the reconstituting membrane system after imbibition, the cell wall-plasma membrane continuum, and changes in intracellular physical states (Ma et al., 2026). The seed coat and outer mucilage are the first interfaces that salt, water, and high pH encounter before reaching embryonic tissues, and serve primarily as buffers and diffusion regulators (Şekerci et al., 2025; Ma et al., 2024). In *Arabidopsis*, *ERECTA* family receptor kinases regulate germination initiation under salt and osmotic stress by influencing mucilage layer differentiation and expansion, accompanied by altered expression of germination repressors such as *ABI3*, *ABI5*, *RGL2*, and *DOG1* (Nanda et al., 2019), suggesting that tissues surrounding the embryo contribute to salt-induced germination delay. In the extreme halophyte *Schrenkiella parvula*, salt-induced changes in mucilage structure are associated with better germination performance and differential gene expression (Şekerci et al., 2025), implying a possible role of mucilage in adaptation to saline environments in halophyte seeds. However, most of this evidence comes from model plants or halophytes and addresses neutral salt or osmotic stress. Direct evidence for the role of mucilage in buffering ion entry and high pH in crop species under combined saline-alkali conditions remains limited.

After imbibition, membrane system reconstruction is an important component of early responses. During water uptake by dry seeds, membrane lipids must transition from a leaky state to a selectively permeable state (Shi et al., 2022). Under salt or saline-alkali stress, membrane repair, Na^+^ influx, and ROS production may act in combination (Rasheed et al., 2022). In *Ricinus communis*, ZnO nanoparticle treatment improved germination rate and reduced malondialdehyde (MDA) accumulation under NaCl stress. Lipidomic and transcriptomic analyses revealed changes in membrane lipids including phosphatidylcholine (PC), phosphatidic acid (PA), phosphatidylethanolamine (PE), phosphatidylglycerol (PG), and lysophosphatidylcholine (LPC), maintenance of lipid unsaturation, conversion of lipids to carbohydrates, and mitogen-activated protein kinase (MAPK)-related pathways (Han et al., 2025). These observations link improved germination under salt stress to membrane lipid homeostasis, antioxidant protection, and metabolic reprogramming of reserves, but do not establish any particular lipid molecule as a universal salt sensor. In aged *Avena sativa* seeds, the phospholipase C–diacylglycerol kinase (PLC–DGK) pathway and membrane lipid remodeling during germination occurred within a defined temporal window and were associated with membrane repair and seed vigor maintenance (Yi et al., 2025). Although this system did not involve saline-alkali stress, it suggests that early membrane lipid remodeling during imbibition may provide a rapid regulatory layer.

The cell wall-plasma membrane continuum may translate mechanical changes caused by salt, high pH, and low water potential into Ca²^+^ signals and cell wall remodeling responses (Qin et al., 2026). FERONIA (FER) and related CrRLK1L receptor kinases serve as a paradigm for this process (Liu et al., 2024). Under salt stress, FER interacts with pectin through its extracellular malectin-like domain and triggers cell-type-specific Ca²^+^ transients to maintain cell wall integrity; *fer* mutants showed root cell rupture upon recovery after salt stress (Feng et al., 2018). Pectin methylesterase activation and cell wall modification also depend partly on the coordinated action of receptor kinases including FER, HERK1, and THE1 (Gigli-Bisceglia et al., 2022). These results are derived mainly from root tissues at the seedling stage and cannot be directly extrapolated to radicle protrusion mechanisms during germination. At the seed level, FER participates in ABA-mediated germination regulation through interaction with CARK1 and modulation of ABI5 stability (Wang et al., 2024c). The CBL5-CIPK8/CIPK24-SOS1 module is involved in NaCl signal transduction and promotes Na^+^ efflux during *Arabidopsis* seed germination; *cbl5* mutants were severely inhibited in germination under salt stress, whereas *CBL5* overexpression restored germination (Xie et al., 2024). This suggests that early perception during germination may be coupled to ion extrusion capacity.

Liquid-liquid phase separation (LLPS) represents a candidate mechanism for rapid osmotic perception after imbibition. As seeds transition from the glassy state to the hydrated state, cytoplasmic viscosity, molecular crowding, ionic strength, and water potential all change. Salt stress further restricts water uptake, and protein condensates may participate in sensing water status. In *Arabidopsis*, FLOE1 undergoes hydration-dependent phase separation during imbibition, and its condensation state modulates germination inhibition under water stress (Dorone et al., 2021). The permeability of the seed coat lignin barrier, mediated by LAC3/5/12/13, affects FLOE1 phase separation and germination rate under osmotic stress (Zhuang et al., 2025). In *Oryza sativa*, OsBTZ1/3 form dynamic condensates and recruit OsSnRK1αB under NaCl conditions, and the *osbtz1/3* double mutant showed reduced germination rate under salt stress (Li et al., 2025a). However, these findings mainly involve water stress, osmotic stress, or NaCl conditions, and cannot yet be extrapolated to high-pH saline-alkali combined stress. Thus, LLPS is currently more appropriate as a candidate regulatory module for saline-alkali tolerance during germination rather than as a fully validated core pathway.

### 3.2 Ion and redox homeostasis

Whether a seed can continue to germinate depends on its ability to rapidly reestablish internal homeostasis to support growth. Ionic homeostasis during germination involves not only Na^+^ efflux, but also restriction of Na^+^ influx, K^+^ retention, restoration of proton motive force, maintenance of membrane integrity, and ROS scavenging. During early imbibition, membrane lipids and proteins are still being restored, so salt entry and high pH interference tend to manifest first as membrane system instability. Ionic imbalance in turn promotes ROS accumulation, and excess ROS further induces membrane lipid peroxidation and impairs transporter function. Germination failure, therefore, is generally not the result of ionic toxicity alone, but rather a consequence of ionic and oxidative damage not being contained within a limited time window.

Components of the Salt Overly Sensitive (SOS) pathway affect not only seed germination and seedling establishment under salt stress, but also H_2_O_2_ scavenging capacity in *Arabidopsis* (Yang et al., 2025), suggesting that the classical Na^+^ homeostasis pathway and redox recovery are not independent of each other. In *Zea mays*, *ZmHAK17* is upregulated in the germinating embryo, particularly in the radicle, under salt stress, promotes Na^+^ efflux from the radicle, and limits excessive Na^+^ accumulation in the germinating embryo; its loss enhances salt sensitivity during germination (Wang et al., 2024b). In *Arabidopsis*, Ca²^+^ supplementation improves germination rate and K^+^ retention under salt stress through the HKT1;1/PP2C49-related network (Chandran et al., 2024). These findings indicate that ionic homeostasis during germination does not occur uniformly across the entire seed, but may preferentially take place in key tissues such as the radicle.

Comparative studies across crop species have shown that salt tolerance during germination is generally determined by multiple homeostatic pathways acting together. In *Triticum aestivum* under NaCl stress, different salt-tolerant genotypes exhibited distinct patterns of Na^+^, K^+^, and P accumulation, antioxidant enzyme activities, and expression of *TaAVP1* and *NHX1* (Sallam et al., 2026). Salt tolerance at the germination stage is therefore better characterized as the combined outcome of ion exclusion, K^+^ retention, ROS buffering, and membrane functional recovery. It should be noted, however, that most direct evidence currently available comes from neutral salt treatments; the additional effects of high pH on transmembrane gradients, transport activity, and redox balance during the seed stage remain poorly resolved.

### 3.3 Autophagy and proteostasis

Restoration of ion and ROS homeostasis does not necessarily drive germination forward. Seeds after imbibition must also remove oxidative damage accumulated during storage, as well as damaged proteins and abnormal organelles generated during metabolic reactivation (Haq et al., 2025). Autophagy maintains protein and organelle homeostasis through removal, recycling, and reuse, and provides substrates for membrane repair, new protein synthesis, and early growth (Iglesias-Fernández and Vicente-Carbajosa, 2022). For germinating seeds that have not yet established stable external uptake capacity, this process may be an important component of homeostatic reestablishment.

Direct evidence at the seed level comes from *Arabidopsis*. Autophagy-deficient mutants showed significantly reduced germination ability after prolonged storage; removal of the seed coat and endosperm allowed the embryos to continue developing, indicating that impaired germination was not due solely to insufficient embryonic activity but was associated with reduced endosperm quality (Shinozaki et al., 2024). Defective autophagy also led to increased oxidized protein accumulation and cell death in the endosperm, and weakened expression of genes involved in endosperm cell wall degradation and remodeling, including *ENDO-β-MANNANASE 7* and *EXPANSIN 2* (Shinozaki et al., 2024). These observations link autophagy to oxidative damage control and plasticity of the enclosing tissues. Salt stress enhances ROS accumulation, protein oxidation, and membrane system stress, so autophagy may participate in damage buffering and resource reallocation during germination under salt stress. However, studies directly addressing autophagy flux in the context of saline-alkali stress during seed germination remain limited. Thus, autophagy is currently more appropriate as a mechanistic clue for protein and organelle homeostasis restoration than as a fully validated core pathway for saline-alkali germination.

### 3.4 Cell wall biomechanics and radicle protrusion

Homeostatic reestablishment must ultimately translate into radicle protrusion. Seed germination is not only a consequence of metabolic recovery and changes in gene expression, but also a process of rebalancing embryonic growth potential against the mechanical resistance imposed by enclosing tissues such as the seed coat and endosperm (Steinbrecher and Leubner-Metzger, 2017). Endosperm weakening is a prerequisite for radicle protrusion in most angiosperms, involving expansins, cell wall hydrolases, apoplastic ROS, cell wall loosening, and local cell separation (Zhang et al., 2025a). Under saline-alkali conditions, even if the embryo retains growth potential, germination may still stall if endosperm weakening is delayed or if radicle turgor and penetration force are insufficient.

In *Arabidopsis*, *AtEXP2* is expressed specifically in germinating seeds and is regulated by the GA/DELLA module; the *exp2* mutant showed delayed germination and increased sensitivity to salt and osmotic stress, whereas overexpression lines exhibited enhanced tolerance (Yan et al., 2014). Overexpression of *BoEXPA2* in *Brassica oleracea* var. *acephala* also improved germination rate under salt and osmotic stress, accompanied by higher antioxidant enzyme activities and lower MDA levels (Sun et al., 2024). These results connect cell wall loosening proteins to hormonal regulation, antioxidant status, and radicle protrusion. In *Solanum lycopersicum*, the ABA import transporter *AIT1.1* is expressed mainly in the endosperm after imbibition. By transporting ABA into the micropylar endosperm cells, it suppresses the expression of genes involved in endosperm weakening, thereby restricting radicle protrusion under high-salt conditions; the *ait1.1* mutant showed faster radicle emergence and higher germination rate under salt stress (Shohat et al., 2023). This suggests that the enclosing tissues are not a passive resistance layer, but rather an interface where hormonal action and mechanical constraints are integrated under salt stress.

**Figure 3:**
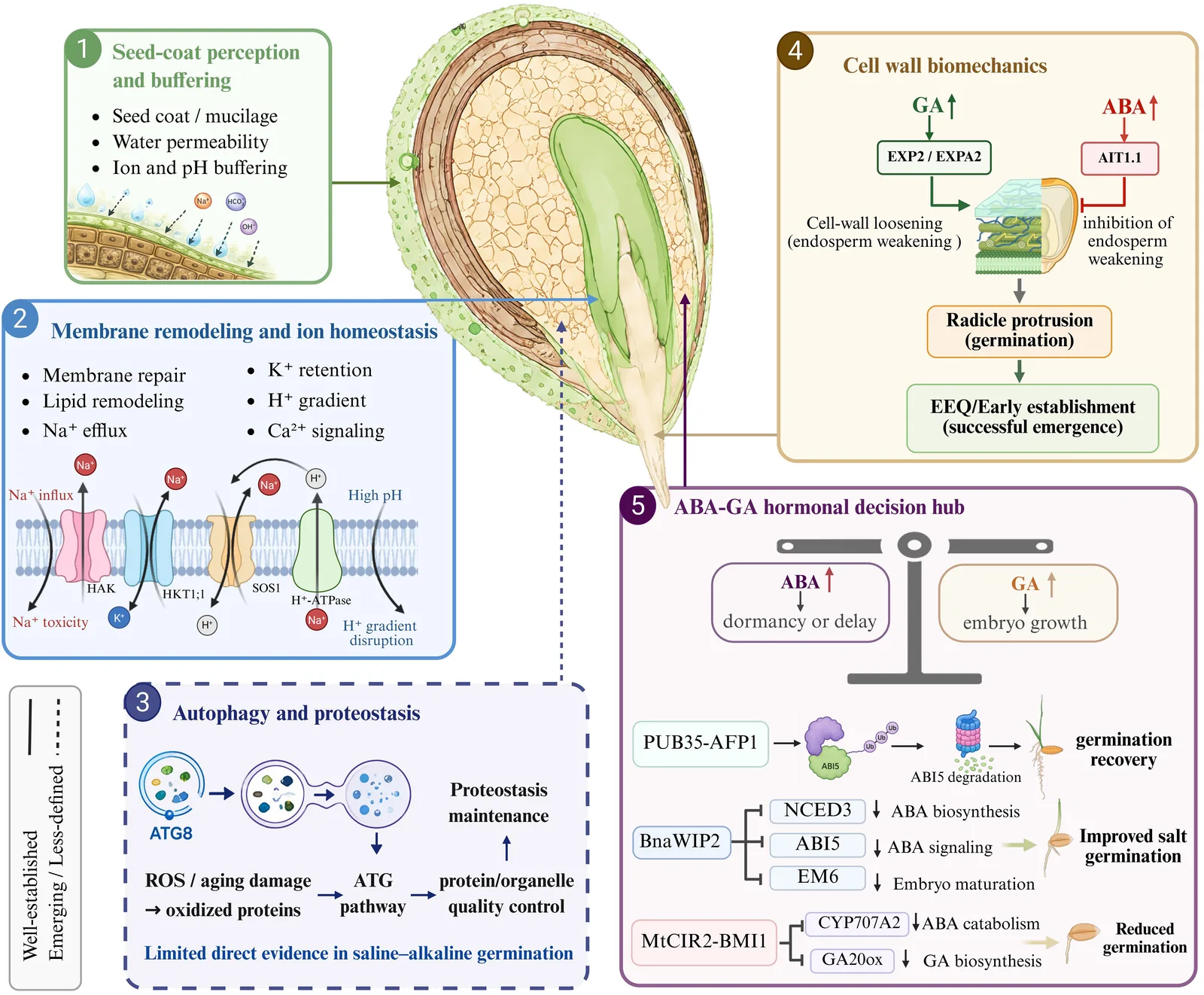
Regulatory modules underlying seed germination under saline-alkali stress. Five interacting modules are shown: (1) seed-coat perception and buffering; (2) membrane remodeling and ion homeostasis; (3) autophagy and proteostasis; (4) cell wall biomechanics; and (5) ABA–GA hormonal decision hub. Coat and mucilage traits may affect water entry, ion diffusion and local pH buffering. Membrane repair, Na^+^ efflux, K^+^ retention, H^+^ gradients and Ca²^+^-related signaling support early cellular homeostasis under salt and high-pH pressure. Autophagy and proteostasis is included as an emerging theme for quality control of oxidized proteins and damaged organelles; direct evidence in saline-alkali germination remains limited. Cell-wall loosening and endosperm weakening set a mechanical threshold for radicle protrusion, and ABA-GA balance integrates stress into dormancy/delay versus embryo growth. Selected genes and proteins are illustrative examples from diverse species and stages, not a single universal pathway. Solid versus dashed styling indicates better-supported versus emerging/less-defined relationships; question marks denote hypotheses requiring validation.

Cell wall remodeling is not necessarily stronger or more extensive. In *Suaeda liaotungensis*, SlXTH1 is strongly expressed in the radicle and hypocotyl of germinating seeds. However, when overexpressed in *Arabidopsis*, SlXTH1 increased xyloglucan endotransglucosylase (XET) activity and altered xyloglucan and cellulose content, but reduced germination rate under salt stress, accompanied by elevated H_2_O_2_ and MDA levels and decreased antioxidant enzyme activities (Song et al., 2025). Studies in *Lepidium sativum* also showed that non-optimal temperatures can inhibit endosperm cap weakening through distinct molecular and biomechanical mechanisms (Steinbrecher et al., 2025). Thus, saline-alkali germination requires controlled wall rearrangement rather than indiscriminate modification of wall components. The regulatory modules underlying saline-alkali germination are summarized schematically in **Figure 3**.

## 4 The growth-defense balance

### 4.1 The ABA-GA hub

Under saline-alkali stress, germination is not about stronger defenses being more beneficial; rather, it requires coordinating damage control with growth progression under limited reserves (Ghosh et al., 2025). Seeds have not yet established a stable photosynthetic supply, yet they must accomplish imbibition recovery, reserve mobilization, ion and redox buffering, and radicle protrusion (Hualpa-Ramirez et al., 2024). Excessive defense delays germination, whereas premature growth increases the risk of damage. At this decision point, the ABA-GA balance provides a relatively robust explanatory framework. ABA generally maintains dormancy or delays germination, whereas GA promotes reserve mobilization, cell expansion, and radicle elongation (Xiao et al., 2026a). The relative state of these two hormones, rather than the absolute content of either alone, determines whether a seed remains arrested or continues to grow (Zhao et al., 2022). Salt stress often shifts the system toward ABA dominance (Sun et al., 2025), thereby suppressing germination-related metabolism and radicle growth. Germination may resume only when GA action is restored or ABA inhibition is alleviated (Ghosh et al., 2025).

ABI5 is an important output node of this balance. As a key transcription factor mediating ABA-induced germination inhibition, ABI5 integrates ABA, GA, and light signals and influences the germination window (Zhao et al., 2022). FER can promote ABA-induced SnRK2 activation and ABI5 accumulation through regulation of CARK1 kinase activity, thereby enhancing ABA inhibitory responses during early germination (Wang et al., 2024c). However, that study was not conducted under saline-alkali stress and is more appropriately viewed as a mechanistic reference for ABA signaling in germination regulation. In contrast, PUB35 promotes ubiquitination and degradation of ABI5 through AFP1; the *pub35* mutant showed increased sensitivity to ABA, salt, and osmotic stress (Du et al., 2024), suggesting that ABI5 turnover affects the reversibility of germination inhibition.

Salt stress can also reshape the ABA-GA balance at the transcriptional and epigenetic levels. In *Brassica napus*, *BnaWIP2* is induced by NaCl and ABA during early germination; its overexpression promotes germination under salt stress, whereas knockout reduces germination. Mechanistically, *BnaC06.WIP2* suppresses the expression of genes involved in ABA biosynthesis or signaling, including *NCED3*, *ABI5*, and *EM6*, thereby limiting excessive ABA accumulation (He et al., 2025). In *Medicago truncatula*, salt-induced lncRNA *MtCIR2* interacts with BMI1, a component of PRC1, and enhances H2A ubiquitination at the *CYP707A2* and *GA20ox1/2* loci, which suppresses ABA catabolism and GA biosynthesis, leading to reduced germination rate under salt stress (Sun et al., 2026). In addition, GA can trigger ATG8-dependent autophagic degradation of DELLA proteins under nutrient starvation and darkness, promoting germination and early seedling establishment (Zhang et al., 2025b). This evidence is not derived from salt stress experiments, but suggests a possible link between hormonal output and autophagy-mediated homeostatic restoration.

### 4.2 Fine-tuning by other hormones

The ABA–GA axis constitutes the core regulatory framework for germination, but its effects are modulated by other hormones and hormone-like factors (Ghosh et al., 2025). Ethylene, brassinosteroids (BR), jasmonic acid (JA), salicylic acid (SA), auxin, and melatonin can all influence germination and early establishment through interactions with ABA or GA. However, the direction of these effects depends on species, dosage, treatment method, and stress intensity, and the overall strength of evidence remains lower than that for the ABA-GA axis (Yu et al., 2020).

Melatonin is a relatively common exogenous regulator in studies of germination under salt stress, with reported effects involving antioxidant defense, ion homeostasis, reserve mobilization, GA biosynthesis, ABA catabolism, and Ca²^+^ signaling (Wang et al., 2024a). In *Sophora alopecuroides*, melatonin alleviated the inhibition of germination and subsequent growth caused by combined neutral salts (NaCl/Na_2_SO_4_) accompanied by reduced ROS, H_2_O_2_, and MDA levels and enhanced antioxidant systems (Zhang et al., 2026d). In *Glycine max*, melatonin improved germination and seedling establishment under NaCl stress, as reflected by enhanced antioxidant and osmotic adjustment capacity, decreased ABA content, and increased IAA, GA, and JA levels. However, higher doses did not confer additional benefits, indicating a dosage window for exogenous application (Zhang et al., 2026e). Brassinosteroids and ethylene may serve as finer modulatory layers. In *Atractylodes macrocephala* Koidz, 24-epibrassinolide alleviated NaCl-induced inhibition of germination and early growth, with associated improvements in hormonal regulation, sugar metabolism, and radicle vigor (Zhang et al., 2024a). Ethylene is involved in seed germination, root establishment, and salt stress adaptation (Zhang et al., 2026c), and interacts extensively with ABA, GA, JA, and BR (Waadt et al., 2022). However, many of these studies include seedling stages and do not yet provide sufficient evidence for an independent causal role of these hormones in saline-alkali stress specifically at the germination stage.

### 4.3 Halotropism as an escape strategy

Beyond internal homeostatic regulation, spatial heterogeneity of salt may also allow the radicle to benefit from directional avoidance. Halotropism can be viewed as an external escape strategy that operates in parallel with internal detoxification mechanisms such as ion efflux and compartmentation (Korver et al., 2020; Mazumder et al., 2025). The strongest evidence currently available comes from seedling primary roots. In *Arabidopsis*, tomato, and sorghum, roots curved away from the gravity direction toward the low-salt side under a NaCl gradient, driven by asymmetric auxin distribution in the root tip resulting from enhanced *PIN2* endocytosis on the high-salt side (Korver et al., 2020). Subsequent studies further indicated that root tip architecture and remodeling of the auxin reflux loop are important cellular bases for halotropism (van den Berg et al., 2016). The root cap-localized NAC transcription factor *SOMBRERO* promotes root avoidance of high-salt zones by maintaining *AUX1* expression in the lateral root cap and spatiotemporal auxin redistribution, and this effect is not a simple consequence of changes in cellular Na^+^ balance (Zheng et al., 2024).

These conclusions, however, cannot be directly extrapolated to early germination. The available evidence comes mainly from seedling primary roots days after germination, when root tip architecture is already well established. During radicle protrusion, the embryo is still constrained by the seed coat and endosperm, and root cap function, cell wall mechanics, and directional bending capacity may not yet be fully developed. A more cautious interpretation is therefore that seedling roots possess a clear salt-avoiding bending mechanism, whereas whether the radicle executes a complete halotropic program during early germination awaits verification through dynamic imaging and genetic evidence. Nevertheless, halotropism merits inclusion in the emergence framework, because salt distribution in surface soils is often patchy, and the direction of early growth after radicle protrusion may influence rooting and survival probability.

## 5 Cross-stage integration and the SEC framework

### 5.1 Epigenetic memory and transgenerational effects

Germination under saline-alkali stress is not an isolated short-term event. The hormonal state, reserve mobilization efficiency, ROS scavenging capacity, and initial transcriptional status established during germination may continue to influence seedling response thresholds to salinity after radicle protrusion. In halophyte *Limonium irtaense*, seeds whose germination was inhibited by NaCl recovered when transferred to salt-free conditions, and seedlings derived from salt-treated seeds showed higher survival rates and biomass under subsequent salt treatment (Ferriol et al., 2026). In *Oryza sativa*, cyanophycin treatment improved germination rate under salt stress and enhanced α-amylase activity, osmotic regulation, and antioxidant metabolism, with effects extending to the seedling stage (Zhou et al., 2026). However, this continuity does not mean that germination salt tolerance can directly predict tolerance at the seedling or whole-plant stage, as different species still exhibit marked differences in germination thresholds, Na^+^ partitioning, antioxidant responses, and transcriptional regulation (Gillani et al., 2026).

Delayed or reduced germination during salt stress should not automatically be interpreted as physiological failure. In halophytes and some stress-adapted taxa, at least four situations must be distinguished: irreversible loss of viability, reversible osmotic inhibition (Zhang et al., 2024b), dormancy or quiescence (Kazachkova et al., 2016), and recovery germination after stress relief (Debez et al., 2018). In such cases, low GP or slow germination under salt stress may reflect adaptive delay rather than weak tolerance, provided that viability is retained and germination resumes when conditions improve. For crop screening aimed at rapid and uniform establishment, delayed germination under stress is generally unfavorable; for ecological interpretation of halophyte adaptation, however, recovery behavior may be more informative than stress-phase kinetics alone.

Epigenetic regulation provides a possible mechanism for this continuity. Loss of the m^6^A methyltransferase OsMTA1 in *Oryza sativa* delayed germination and exacerbated oxidative damage under salt stress, accompanied by altered m^6^A modification and expression of genes involved in germination, growth, and stress responses (Li et al., 2025c). In *Arabidopsis*, the histone deacetylase AtSRT2 regulates salt tolerance during seed germination; its loss of function leads to salt hypersensitivity, accompanied by altered histone acetylation at stress-responsive loci (Tang et al., 2022). More persistent salt-induced states have been observed mainly at post-germination stages. Transcriptional memory of *P5CS1* is associated with H3K4me3 retention during recovery and with *HY5* regulation (Feng et al., 2016). Mild salt treatment (priming) of *Arabidopsis* seedlings can establish somatic stress memory, which is accompanied by changes in both H3K27me3 and H3K4me3 profiles at specific stress-responsive genes, such as *OPR3*, *AOC1*, and *LOX3* (Arıkan et al., 2026). DNA methylation is often incorporated into broader frameworks of stress memory and transgenerational inheritance (Sun et al., 2022). Thus, m^6^A, H3K4me3, H3K27me3, and DNA methylation provide mechanistic clues for how germination experience may influence subsequent adaptation, but direct evidence that saline-alkali exposure during germination stably persists to the seedling stage remains limited.

Transgenerational effects also require cautious interpretation. In *Solanum lycopersicum*, seeds subjected to polyamine priming maintained enhanced salt tolerance after long-term storage, and higher antioxidant and osmotic adjustment capacity was also observed in the progeny (Vicente et al., 2025). In *Oryza sativa*, maternal exposure to saline field conditions and treatment of mother plants with wood vinegar or spermidine improved early germination rates of progeny seeds under salt stress, accompanied by changes in polyamine and H_2_O_2_ status (Nounjan and Theerakulpisut, 2023). Studies in *Triticum aestivum* (Thabet et al., 2025) and *Hordeum vulgare* (Kakar et al., 2025) also suggest that parental salt stress experience may influence progeny germination, antioxidant capacity, and certain agronomic traits. In *Arabidopsis*, CeO_2_ nano-priming was associated with DNA methylation changes and conferred partial salt tolerance to progeny seedlings (Li et al., 2025b). However, transgenerational effects are not equivalent to epigenetic memory; maternal nutrition, seed maturation environment, and short-term physiological carryover may all contribute.

### 5.2 The sequential emergence capacity framework

**Figure 4:**
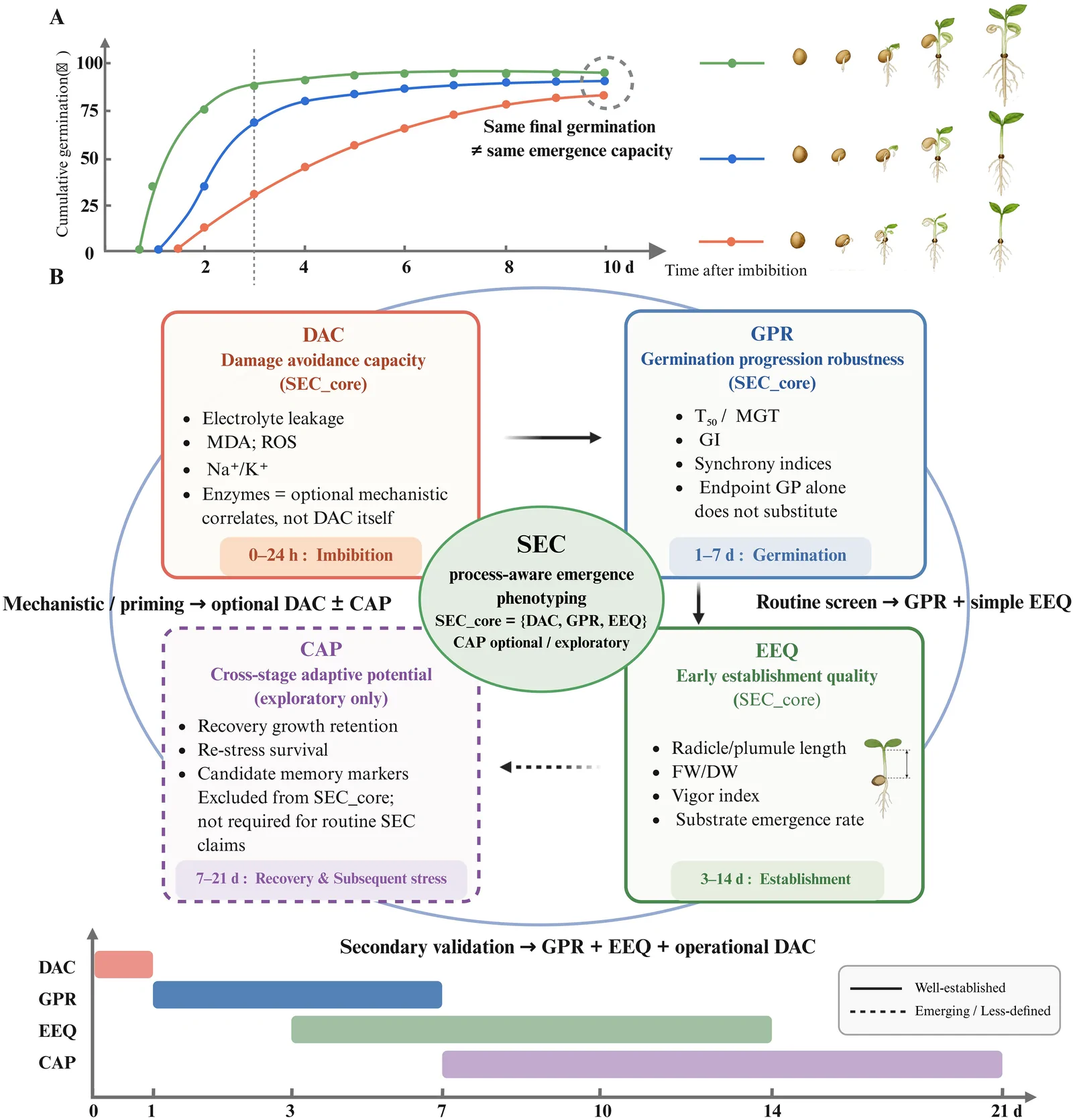
Sequential emergence capacity (SEC) under saline-alkali stress. **(A)** Schematic germination trajectories showing that identical final GP can correspond to different kinetics, injury loads, and early-establishment prospects. Curves are schematic and do not represent a specific dataset. GP, germination percentage; T50, time to 50% germination; MGT, mean germination time; GI, germination index. **(B)** Operational SEC framework. Solid boxes indicate SEC_core = {DAC, GPR, EEQ} (damage avoidance capacity; germination progression robustness; early establishment quality) along approximate observation windows; the dashed box indicates CAP (cross-stage adaptive potential) as exploratory only, not required for routine SEC claims. Brief metric lists are shown for each dimension; optional enzymatic readouts serve as correlates but are not part of DAC itself. Side annotations indicate practical tiers, such as routine screening, secondary validation, or mechanistic and priming studies. Default reporting is a multidimensional profile rather than a single universal score. SEC complements final GP and classical vigor indices; it is not a new molecular tolerance mechanism.

In saline-alkali environments, GP and GE remain fundamental indicators, but they primarily reflect endpoint outcomes and cannot reveal kinetic delays, changes in synchrony, hidden injury load, or the quality of early establishment after radicle protrusion (Manono, 2026). Classical seed vigor testing already targets uniform germination and competent early seedling growth, which is the agronomic foundation of stand establishment. It is commonly summarized by germination speed and synchrony metrics, seedling growth, and vigor indices alongside GP and GE. Methodologically, the idea that identical final germination percentages can hide differences in speed, timing, and spread is already well established in seed science. Kader (2005), for example, systematically compared final GP with MGT, GI, coefficient of velocity of germination, germination rate index, first and last days of germination, and time spread of germination, and showed that final GP alone is insufficient because different indices capture different aspects of the process. Under combined saline-alkali or alkaline stress, reductions in radicle length, fresh weight, dry weight, and vigor index are often greater than reductions in GP or GE (Zhang et al., 2024b; Deng et al., 2020). Continuous germination analysis in *Oryza sativa* also indicated that salt stress not only reduces maximum germination potential but also delays the time to reach maximum germination rate and prolongs the germination period (Kruthika and Jithesh, 2023). Thus, the same final GP may correspond to different germination trajectories and emergence prospects (**Figure 4A**). Relying solely on final GP therefore risks underestimating progressive damage and subsequent establishment failure.

To address this limitation, we propose sequential emergence capacity (SEC). It is proposed as a process-oriented complement to conventional endpoint indicators (**Figure 4B**). It asks whether seeds can complete the ordered transition from imbibition and metabolic reactivation to radicle protrusion and early establishment under saline-alkali stress while keeping early injury within recoverable limits. SEC does not redefine saline-alkali tolerance as a new molecular pathway, nor does it replace GP, GE or classical vigor indices. Its contribution is operational: it decomposes the emergence window into early injury load, progression and post-protrusion establishment; assigns these readouts to different testing tiers; and requires external anchoring whenever a scalar score is used.

SEC comprises four dimensions (**Table 1**). The first is damage avoidance capacity (DAC) quantifies early injury load during imbibition and metabolic reactivation, before radicle protrusion. Primary operational readouts include electrolyte leakage, MDA, ROS/H_2_O_2_ and tissue Na^+^/K^+^ (Hualpa-Ramirez et al., 2024; Balasubramaniam et al., 2023). Antioxidant enzyme activities and imaging-based assays can help explain a low injury load, but they are mechanistic correlates rather than the DAC score itself.

**Table 1:** Optimized four-dimensional framework of sequential emergence capacity (SEC) under saline-alkali stress.

SEC dimension	Evaluation focus	Recommended tier	Core metrics	Mechanistic/extended metrics	Recommended window	Key constraints
DAC (SEC_core)	How severe is early injury load during imbibition and metabolic reactivation?	Secondary validation; Mechanistic validation	Electrolyte leakage; MDA; ROS/H_2_O_2_ level; Na^+^/K^+^ ratio	Membrane-integrity staining; SOD/CAT/APX/POD activities; tissue-specific ion distribution	0-24 h after imbibition; before radicle protrusion	Mostly destructive; costly; highly time-sensitive; enzymes explain mechanism but are not DAC itself
GPR (SEC_core)	How fast, synchronous, and delay-resistant is germination under stress?	Routine screening	T_50_; MGT; GI; Synchrony-related indices	T_10_/T_90_; AUC; Logistic/Gompertz parameters	1-7 d after sowing; species-dependent	Sensitive to culture system and scoring criteria; endpoint GP alone is insufficient
EEQ (SEC_core)	Is radicle protrusion converted into viable early establishment?	Routine screening; Secondary validation	Radicle/plumule length; fresh/dry weight; vigor index; substrate emergence rate and uniformity	Survival rate; root-architecture traits	3-14 d after radicle protrusion	Strongly affected by substrate, moisture, temperature, and mechanical resistance
CAP (Exploratory only)	Do germination-stage experiences or priming alter later recovery/re-exposure performance?	Mechanistic validation; Exploratory dimension	Recovery growth retention rate, survival rate or growth retention under re-exposure	Ion/ROS recovery rate; ABA/GA status; candidate epigenetic/memory markers	7-21 d after germination; monitor recovery or re-exposure	High cost, long cycle; difficult to separate memory from carryover effects

SEC, sequential emergence capacity; SEC_core, operational core of SEC (DAC + GPR + EEQ). CAP is exploratory and should not be required for routine SEC reporting. Primary operational metrics are intended for comparative phenotyping; optional mechanistic metrics support interpretation in smaller experiments. The metrics listed under GPR are candidates; a non-redundant, biologically complementary subset should be selected according to the experimental objective. SEC is a process-oriented complement to final GP, GE, and vigor indices, not a replacement. Weighting schemes, if used, must be crop-, stress-, and system-specific and ideally anchored to substrate- or field-level emergence. Neutral-salt and alkaline-salt (or pH-matched) treatments should be reported separately. Under alkaline salts, optional additional readouts include medium pH, early radicle/hypocotyl sensitivity to high pH, and early phenotypes linked to nutrient availability. The dimensional structure of SEC may be generalizable, but biological interpretation and any weights must not be transferred across salt types without re-validation. MDA, malondialdehyde; ROS, reactive oxygen species; SOD, superoxide dismutase; CAT, catalase; APX, ascorbate peroxidase; POD, peroxidase; MGT, mean germination time; GI, germination index; AUC, area under the curve; GP, germination percentage.

The second is germination progression robustness (GPR) captures speed, synchrony and delay under stress, using complementary kinetic metrics such as T50, mean germination time (MGT), germination index (GI), synchrony-related indices and, where useful, curve-based parameters (Irik and Bikmaz, 2024). Endpoint GP may be reported alongside GPR but does not substitute for progression metrics. The metrics listed under GPR should be viewed as candidate descriptors rather than a set that must all be used together. Many germination variables capture overlapping properties and may be strongly correlated (Debez et al., 2018). Including too many of them can overrepresent speed-related information and introduce collinearity or double weighting. For most applications, a minimal set of biologically complementary metrics is preferable (Sedghi, 2025). For example, one metric of final germination capacity, one for germination speed (such as T50 or MGT), one for temporal spread or synchrony, and one early-establishment metric from EEQ would often suffice. The choice of variables should depend on the experimental objective. Final capacity, speed, uniformity, recovery, and substrate emergence are related but address different questions.

Low GP or slow germination under saline or alkaline exposure should not be automatically taken as physiological failure or inherently “poor GPR”. In many halophytes, germination inhibition under high salinity can be an adaptive and reversible strategy that prevents establishment under transiently unfavorable conditions (Şekerci et al., 2026). Therefore, GPR interpretation and any weighting within SEC should depend on the goal. Programs that screen crops for rapid and uniform stand establishment may reasonably penalize delay. In contrast, ecological evaluation of halophytes should not equate stress-induced postponement with low tolerance. Where possible, protocols should combine germination kinetics under stress with viability and recovery assays.

The third is early establishment quality (EEQ), which asks whether radicle protrusion is converted into stable initial structures and stand potential. Core metrics include radicle/plumule length, fresh and dry weight, vigor index, substrate emergence rate, emergence uniformity, and early survival. EEQ is the closest laboratory bridge to agronomic stand establishment.

The fourth is cross-stage adaptive potential (CAP), which examines whether saline-alkali exposure during germination, or seed-priming treatments, alters subsequent seedling performance under recovery or re-exposure. CAP currently has a weaker evidence base, longer experimental cycles, and lower standardization than DAC/GPR/EEQ. It is therefore excluded from SEC_core and recommended only for mechanistic studies, priming validation, or deep characterization of core germplasm. CAP is not required to claim an SEC-based evaluation.

SEC_core = {DAC, GPR, EEQ}, with CAP reported separately when measured.

Because neutral and alkaline salts impose partly distinct constraints, SEC profiles should be reported by regime rather than pooled *a priori* as a generic “saline-alkali” treatment. Weights or thresholds trained under NaCl should not be transferred to alkaline salts without re-validation. For alkaline regimes, medium or substrate pH and early high-pH-sensitive radicle traits are useful interpretive covariates for DAC and EEQ, not an additional SEC dimension (**Table 1**). Any SEC claim should specify crop, salt type, concentration and assay system.

Several lines of evidence in the literature motivated the SEC framework. Endpoint GP can mask divergent kinetics and early seedling vigor under salt/alkali stress. Progression and early growth traits are often more stress-sensitive than final GP. The multi-trait nature of germination performance has a complex genetic basis, with multiple loci and epistasis effects (Zhang et al., 2023). Priming, including classical halopriming with salt solutions in *Raphanus sativus* (Kanjevac et al., 2021) and broader antioxidant-centered priming strategies under salinity (Tolrà et al., 2025), can improve germination speed, uniformity, and early vigor in both model and crop systems.

It is predicted, but not yet validated, that organizing phenotypes into SEC_core or forming any weighted composite will improve prediction of substrate- or field-level emergence rate and uniformity beyond GP (or beyond GP + a single vigor index). Until such external validation is shown for a given crop and stress regime, SEC should be treated as an operational framework for process-aware phenotyping, not as a proven substitute for existing indices.

We propose three minimal testable predictions. Each should be evaluated on independent samples within a defined crop and salt regime. The primary external anchors are substrate- or field-level emergence rate and uniformity.

(H1) When final GP is matched or stratified (fixed-GP comparison), GPR + EEQ still explain residual variation in emergence rate and uniformity beyond GP alone.

(H2) Adding operational DAC improves the identification of “high GP but weak establishment” or fast but unstable trajectories beyond GP/GPR alone, using the same external emergence anchors.

(H3) If a composite SEC score fails to improve emergence prediction over GP or GP + a standard vigor/emergence index on independent samples, that weighting scheme should be discarded for the tested regime.

#### 5.2.1 Added value relative to existing multi-trait approaches

Multi-trait ranking methods, for example PCA, D values, fuzzy evaluation and clustering, have already integrate GP, early growth and vigor traits for germplasm comparison (Chen et al., 2025; Jiao et al., 2024; Hamidi Moghaddam, 2026; Cao et al., 2024). SEC does not claim novelty simply by assembling these traits. Instead, it offers three testable distinctions from endpoint or single-score fusion. First, it uses sequential decomposition along the emergence timeline rather than collapsing traits at one scoring point. Second, it allows tiered use matched to throughput and cost, rather than forcing all data into a single rank (**Tables 1**, **2**). Third, it requires that any optional composite be anchored to substrate- or field-level emergence data. The default SEC output is therefore a multidimensional profile, not a single tolerance score. This is the main conceptual difference from methods that produce a single composite rank as their primary output.

**Table 2:** Practical implementation, scalability, and limits.

Application tier	Recommended dimensions	Typical metrics	Purpose
Routine/high-throughput screening	GPR + simple EEQ	T_50_/MGT/GI/synchrony; radicle length; vigor index; substrate emergence and uniformity	Rank large populations with non- or low-destructive readouts
Secondary validation of candidates	Selected DAC operational metrics + GPR + EEQ	Electrolyte leakage and/or MDA and/or Na^+^/K^+^ on targeted samples; refined progression and establishment assays	Test whether superior GP/kinetics coincide with lower early injury load
Mechanistic or priming studies	Optional DAC mechanistic assays ± CAP	Enzyme activities, imaging, recovery/re-exposure, memory markers	Explain mechanisms; validate priming; not for routine ranking

DAC assays are often destructive, time-window-sensitive, and relatively costly; GPR is sensitive to culture system and scoring rules; EEQ depends on substrate mechanical impedance, moisture, and temperature; CAP confounds true memory with carryover injury or residual priming chemicals and is never required for SEC-based ranking. Species, salt composition (neutral versus alkaline), and stress intensity must be reported, because SEC weights and windows are not universal constants. Under alkaline stress, optional readouts include medium pH, early radicle/hypocotyl high-pH sensitivity, and nutrient-availability-related early traits. Dimensional structure may be shared across salt types; interpretation and weights must be re-validated and must not be transferred directly from neutral salt to alkaline salt regimes.

A simple decision rule can guide the use of SEC alongside conventional germination assays. If the only question is whether seeds can germinate under a given regime, GP or GE is sufficient. If the goal is to rank speed, synchrony, and early stand potential, use GPR + EEQ. If the phenotype is high GP but weak establishment, or fast but unstable progression, add operational DAC. Invoke CAP only when the claim concerns durable priming effects, recovery, or re-exposure memory; it is not meant for routine ranking. SEC is not a substitute for evaluating tolerance at the seedling or adult stage.

#### 5.2.2 Optional composite index (non-mandatory)

SEC may be used either as a profile or, optionally, as a composite index when a single scalar is needed for logistical purposes. A composite score is not the default output of SEC and is not required for applying the framework, nor should it be seen as an invitation to pool all available germination metrics. Because many GPR variables are partially redundant, trait selection should come before weighting, with preference given to a minimal set of biologically complementary metrics.

If a scalar score is needed, the dimension scores must first be aligned in direction, normalized within each stress treatment, and standardized. Only then can weights be applied. A general form is:


SEC = α·DAC + β·GPR + γ·EEQ + δ·CAP, α + β + γ + δ = 1

For routine work (δ=0):


SEC_core = α·DAC + β·GPR + γ·EEQ

CAP is reported separately when measured. If DAC is not assayed, any composite should be limited to GPR and EEQ rather than imputing missing injury data. A weighting scheme should not be adopted for a given crop-stress regime unless it improves emergence prediction at the substrate or field level over GP alone, or over GP combined with a standard vigor or emergence metric. If a composite is constructed, Weight sources must be reported transparently and benchmarked against GP-only and GP + vigor baselines. Without such anchoring, composite scores risk reproducing undifferentiated multi-trait stacking under a new name.

### 5.3 Translational pathways

Translating saline-alkali tolerance at the germination stage into stable emergence performance can be advanced through three routes: molecular breeding, seed priming, and microbiome engineering. These routes should be aligned with SEC dimensions rather than with a single endpoint GP. Molecular breeding targets should prioritize tissues and processes that directly affect emergence, including the seed-coat interface properties, early radicle ion homeostasis (Zhu et al., 2025), ABA-GA decision nodes (Ghosh et al., 2025), and, where relevant, memory-related factors (Albaladejo-Marico et al., 2025). GWAS and functional studies already indicate that some loci affect germination rate and post-germination primary-root establishment under salt without necessarily matching whole-plant salt tolerance (Guan et al., 2024; Zhang et al., 2023). Mapping should therefore be performed trait-wise within SEC_core (progression loci vs early-injury loci vs establishment loci), and radicle- or seed-coat-preferential expression strategies may reduce whole-plant growth penalties relative to constitutive overexpression (Wang et al., 2025a).

Seed priming remains the most practice-proximal intervention for the germination window. Beyond newer treatments such as carbon nanodots (Zhang et al., 2026a), GABA (Wei et al., 2024), melatonin (Duan et al., 2025), selenium- and zinc-based nanomaterials (Saber et al., 2023; Islam et al., 2026), and cold plasma (Shahabi et al., 2025), classical halopriming has long been shown to improve salt-stressed germination and early vigor. For example, priming *Raphanus sativus* seeds with moderate CaCl_2_, KCl, or NaCl before exposure to saline conditions significantly improved GP, rate, and uniformity, as well as seedling growth and vigor relative to direct salt exposure, with salt-type- and dose-dependent optima (Kanjevac et al., 2021). Parallel lines of evidence on antioxidant-linked priming under salinity further support redox pre-conditioning as a recurring route to more stable early performance (Tolrà et al., 2025). Within SEC, successful priming should be judged not only by higher final GP, but by lower early injury load (DAC operational metrics where measured), improved GPR (shorter T_50_/MGT, higher synchrony), and stronger EEQ (emergence uniformity and early biomass). CAP-type recovery/re-exposure assays may be added when the claim concerns durable preconditioning rather than immediate germination gain. Critically, priming efficacy remains contingent on species, dose, salt composition (neutral vs alkaline), seed lot quality, and production environment; laboratory gains should be treated as candidate benefits until verified under substrate or field saline-alkali conditions.

Microbiome engineering offers a complementary route by constructing a protective local microenvironment around the seed to improve germination and early rooting. Endophytes (Li et al., 2025d) and plant growth-promoting rhizobacteria (PGPR) (He et al., 2026) can improve germination and early rooting under salt via ACC deaminase-mediated ethylene buffering, exopolysaccharide-mediated interfacial protection, and modulation of antioxidant and hormone metabolism (Xu et al., 2026). This route is closer to real soils but constrained by colonization stability, host-soil compatibility, coating survival, and cost. A realistic translational stack is therefore: genetic improvement for basal SEC_core performance, priming to stabilize the germination window, and microbiome tools to buffer the seed-soil interface. Synergy among the three is plausible but still largely predictive; combinatorial validation against field emergence remains essential.

## 6 Conclusions and future priorities

Saline-alkali tolerance at the germination stage is not an early manifestation of tolerance at the seedling or adult stage, but rather a stage-specific process shaped by early perception, homeostatic reestablishment, growth-defense trade-offs, and cross-stage continuity. Successful emergence in saline-alkali environments depends not only on whether germination initiates, but also on whether seeds can progress steadily through imbibition, membrane repair, ion and redox balance restoration, reserve mobilization, and radicle protrusion.

Three main points emerge from the current literature. First, neutral salt stress and alkaline salt stress should not be simply merged. The former primarily involves ionic toxicity and osmotic stress, whereas the latter imposes additional constraints through high pH, affecting proton-driven processes, the cell wall environment, and nutrient availability. Second, germination progression relies on multilayered homeostatic restoration, with ion homeostasis, ROS buffering, autophagy-related quality control, cell wall mechanics, and the ABA-GA axis jointly influencing radicle protrusion. Third, saline-alkali tolerance during germination cannot be adequately captured by the final GP alone, as the same endpoint may mask different speeds, synchrony, injury loads, and subsequent establishment quality. Process-based phenotyping thus better addresses the emergence problem in saline-alkali fields than any single endpoint measure.

SEC offers a complementary, process-aware phenotyping framework for evaluating saline-alkali emergence, subject to further experimental and field validation. Its operational core, consisting of DAC, GPR, and EEQ, arranges informative readouts along the emergence timeline; CAP remains exploratory. The default output is a multidimensional profile. Any composite score is optional and can be justified only when it improves prediction of substrate- or field-level emergence beyond simpler baselines for a defined crop and salt regime.

Future work should prioritize: (i) spatiotemporal resolution of seed coat, endosperm, and radicle responses during early imbibition; (ii) decoupling neutral-salt from high-pH alkaline effects; (iii) biomechanical measurements during radicle protrusion; (iv) testing whether the early radicle shows halotropic or other directional growth responses; and (v) validating SEC_core profiles, and any composite scores, against substrate and field emergence, with clear reporting of predictive gains over GP-based baselines. Improving emergence on saline-alkali land will depend less on renaming tolerance and more on integrating stage-specific mechanisms, tiered process phenotyping, and field-constrained evaluation.
